# The role of parenting practices in parent and child mental health over time

**DOI:** 10.1192/bjo.2023.529

**Published:** 2023-08-08

**Authors:** Katherine T. Cost, Piyumi Mudiyanselage, Eva Unternaehrer, Daphne J. Korczak, Jennifer Crosbie, Evdokia Anagnastou, Suneeta Monga, Elizabeth Kelley, Russell Schachar, Jonathon Maguire, Paul Arnold, Christie L. Burton, Stelios Georgiades, Rob Nicolson, Catherine S. Birken, Alice Charach

**Affiliations:** Department of Psychiatry, Hospital for Sick Children, Toronto, Canada; and Department of Behavioural Neurosciences & Psychiatry, McMaster University, Canada; Child Health Evaluative Sciences Program, Hospital for Sick Children, Toronto, Canada; University Psychiatric Clinics Basel, University of Basel, Switzerland; Department of Psychiatry, Hospital for Sick Children, Toronto, Canada; and Department of Psychiatry, Faculty of Medicine, University of Toronto, Canada; Department of Pediatrics, Faculty of Medicine, University of Toronto, Canada; and Autism Research Centre, Holland Bloorview Kids Rehabilitation Hospital, Toronto, Canada; Department of Psychology, Queen's University, Canada; and Department of Psychiatry, Queen's University, Canada; Department of Pediatrics, Faculty of Medicine, University of Toronto, Canada; and MAP Centre for Urban Health Solutions, Li Ka Shing Knowledge Institute, Unity Health Toronto, Toronto, Canada; Mathison Centre for Mental Health Research & Education, Cumming School of Medicine, University of Calgary, Canada; and Department of Psychiatry and Medical Genetics, Cumming School of Medicine, University of Calgary, Canada; Department of Psychiatry, Hospital for Sick Children, Toronto, Canada; Department of Behavioural Neurosciences & Psychiatry, McMaster University, Canada; Department of Psychiatry, University of Western Ontario, Canada; Department of Pediatrics, Faculty of Medicine, University of Toronto, Canada; and Division of Paediatric Medicine, Hospital for Sick Children, Toronto, Canada; Department of Psychiatry, Hospital for Sick Children, Toronto, Canada; and Child Health Evaluative Sciences, Hospital for Sick Children, Toronto, Canada; and Department of Psychiatry, Faculty of Medicine, University of Toronto, Canada

**Keywords:** Carers, out-patient treatment, psychosocial interventions, childhood experience, shared parenting

## Abstract

**Background:**

Parent and child mental health has suffered during the pandemic and transition phase. Structured and shared parenting may be intervention targets beneficial to families who are struggling with parent or child mental health challenges.

**Aims:**

First, we investigated associations between structured and shared parenting and parent depression symptoms. Second, we investigated associations between structured and shared parenting and depression, hyperactivity/inattention and irritability symptoms in children.

**Method:**

A total of 1027 parents in two-parent households (4797 observations total; 85.1% mothers) completed online surveys about themselves and their children (aged 2–18 years) from April 2020 to July 2022. Structured parenting and shared parenting responsibilities were assessed from April 2020 to November 2021. Symptoms of parent depression, child depression, child hyperactivity and inattention, child irritability, and child emotional and conduct problems were assessed repeatedly (one to 14 times; median of four times) from April 2020 to July 2022.

**Results:**

Parents who reported higher levels of shared parenting responsibilities had lower depression symptoms (*β* = −0.09 to −0.32, all *P* < 0.01) longitudinally. Parents who reported higher levels of shared parenting responsibilities had children with fewer emotional problems (ages 2–5 years; *β* = −0.07, *P* < 0.05), fewer conduct problems (ages 2–5 years; *β* = −0.09, *P* < 0.01) and less irritability (ages 13–18 years; *β* = −0.27, *P* < 0.001) longitudinally. Structured parenting was associated with fewer conduct problems (ages 2–5 years; *β* = −0.05, *P* < 0.05).

**Conclusions:**

Shared parenting is beneficial for parent and child mental health, even under chaotic or inflexible life conditions. Structured parenting is beneficial for younger children.

The COVID-19 pandemic and transition phase has created unique stressors, particularly for families with children. Very quickly, families lost most of the externally generated daily structure afforded by school attendance, in-person work and recreation,^1^ and had to adjust family life to frequently modified public health requirements. Before the COVID-19 pandemic, around a quarter of families had received childcare support from grandparents, extended family members or befriended parents.^2^ This support was no longer available for many families because of pandemic restrictions. To meet these increased demands, parents needed to engage family management practices to minimise household disruption and reduce dysfunctional family dynamics,^3–6^ especially during transitions between lockdowns and re-openings. Previous literature has described two family management practices associated with improved parent and child well-being:^4^ structured parenting and shared parenting.

## Structured parenting

Structured parenting is a family management approach to provide children and adolescents with predictable and reliable caregiving routines, reasonable limits and scaffolding that correspond to a child's developmental needs.^7^ For example, younger children (ages 2–5 years) may require higher levels of parental monitoring and support in daily instrumental activities, such as personal hygiene, choosing and preparing healthy foods, dressing appropriately for the weather and limiting screen time, in addition to social and affective care. Most older children and adolescents may no longer need high levels of support in daily instrumental activities, so parental caregiving may shift to social, affective and academic support.^7^ In contrast to authoritarian or overprotective parenting,^7^ structured parenting is a flexible and consistent approach to help children and adolescents clarify their roles, increase personal responsibility, accomplish required tasks and develop behaviour regulation.^4^ Relevant to the demands of life during the COVID-19 pandemic, literature published before the pandemic indicates that families with absent or lower levels of structured parenting were more likely to be overwhelmed,^8^ inefficient in meeting the demands of family life,^4^ less adaptable to new challenges^8^ and report higher levels of stress.^9^ Higher levels of structured parenting have also been associated with lower levels of parent and child mental health symptoms in the published literature.^3,5,7–10^

## Shared parenting

Shared parenting refers to the satisfactory division of household and caregiving labour, including joint decision-making and shared parenting time.^11^ Beyond a second parent simply ‘helping’ a primary parent, shared parenting involves both partners taking equal responsibility for household and parenting tasks,^12^ requiring support, coordination and communication between parents.^6^ Parents with higher levels of shared parenting have higher relationship satisfaction,^13^ better family functioning,^4^ lower parental stress,^14^ better child–parent relationships,^15,16^ lower levels of maternal internalising symptoms^14,17^ and better child well-being.^18,19^

## Parent mental health

Given the chronic and variable stresses of COVID-19 pandemic waves, it is unsurprising that parents and children reported deteriorated mental health. A systematic review and meta-analysis on the mental health of mothers with children younger than 5 years reported that 26.9% of mothers met criteria for clinically significant depression symptoms.^20^ Focusing on the mental health of caregivers more broadly, another systematic review and meta-analysis reported that 27.4% of caregivers of children and adolescents up to 18 years of age reported clinically significant depression symptoms.^21^ Importantly, there were no differences in the rate of clinically significant symptoms in depression symptoms between male and female caregivers.^21^

## Child and adolescent mental health

Likewise, several systematic reviews and meta-analyses have indicated that children and adolescents have experienced deteriorations in mental health related to the COVID-19 pandemic. Two systematic reviews on prevalence of clinically elevated depression in children was estimated at 25.2%^22^ and 29.0%,^23^ with both meta-analysis reporting higher symptoms among adolescents compared with younger children. In a meta-analysis that specifically compared child and adolescent depression symptoms before and during the COVID-19 pandemic, symptoms were significantly higher early during compared with before the pandemic, but returned to baseline pre-pandemic levels by late 2020.^24^ In sum, the combination of unique and chronic stressors of the pandemic have had a deleterious impact on parent and child mental health, with potential longer-term consequences for parents and their children.^25–27^ As governments, healthcare organisations and individuals move beyond the acute phase of the pandemic, understanding individual and contextual factors that may contribute to improved parent and child mental health will be key to recovery.

## Theoretical model

Bronfenbrenner's social–ecological model, which is based on concentric circles of growing influence on the child, emphasizes the importance of context in the development of an individual.^28^ At the centre of the model is the individual and their personal characteristics. The individual primarily inhabits the microsystem, including the immediate surroundings, which have the greatest impact on the individual. The microsystem encompasses proximal contexts such as family composition, and more distal ones, including school, paid work and neighbourhood quality. The larger context, conceptualised as the mesosystem, represents relationships, such as relationships with and between family members and interactions among the social–ecological levels. The exosystem includes interactions in community contexts. The macrosystem includes the influences of social, religious and cultural values. Finally, the chronosystem reflects the change over time. Within the context of the COVID-19 pandemic, school closures and lockdowns decreased access to community contexts. As such, different levels within the social–ecological model may have exerted more influence on parent and child well-being. For example, individual factors such as pre-existing mental health diagnoses for the child may have become more salient. Within the macrosystem, home confinement may have resulted in relationships among family members and interactions among the social–ecological levels (balancing parent paid work, online schooling and family demands) having a stronger influence on parent and child well-being. Also, the chronosystem reflecting changes over time in the lockdowns, openings and transition phases (Supplementary Fig. 1 available at https://doi.org/10.1192/bjo.2023.529), as well as changes in child development over 2.25 years, may have presented challenges to parent and child well-being. Our hypotheses are situated within the social–ecological model.

Given that both structured parenting and shared parenting have been associated with better parent and child mental health before the COVID-19 pandemic, we address the evidence gap on the role of structured and shared parenting on parent and child mental health during the pandemic and transition phase – a time of unpredictable stress. Previous literature has examined either structured parenting or shared parenting, without considering both constructs in the same model. Additionally, previous studies are also generally within a specific age group and do not span the breadth of child development. Finally, the examination of structured and shared parenting has often included a limited number of mental health outcomes or general well-being. We aim to identify family management methods that may help to rectify the sustained deterioration in parent^20,21^ and child^22–27^ mental health. Because different developmental periods involve different parenting demands,^29^ we hypothesised that structured and shared parenting may have been important to parent mental health for parents with younger children, but less so for parents with adolescents. Our first objective was to investigate associations between structured parenting and shared parenting with parent depression symptoms longitudinally among parents living in two-parent households with children aged 2–18 years. We hypothesised that both structured and shared parenting would be beneficial for parents’ mental health, with larger effect sizes for parents with younger children than parents with older children and adolescents. Our second objective was to investigate associations between structured and shared parenting, and child depression, hyperactivity/inattention and irritability symptoms longitudinally among children in this same group. We hypothesised that both structured and shared parenting would be beneficial for children's mental health, with larger effect sizes for younger children than older children and adolescents.

## Method

### Participants

This longitudinal cohort study was part of the Ontario COVID-19 and Kids Mental Health Study,^30^ embedded within two clinically referred mental health and neurodevelopmental cohorts and two community cohorts, described fully in our protocol.^30^ By inclusion of both clinical (e.g. children and adolescents with pre-COVID-19 mental health and/or neurodevelopmental diagnoses) and community samples, the impact of the COVID-19 pandemic could be examined across multiple relevant populations.

The authors assert that all procedures contributing to this work comply with the ethical standards of the relevant national and institutional committees on human experimentation and with the Helsinki Declaration of 1975, as revised in 2008. All procedures involving human participants were approved by all institutional research ethics boards (SickKids Research Ethics Board approval number 1000070222; Queen's University Health Sciences Research Ethics Board approval number 6005107; Western University Health Sciences Research Ethics Board approval number 115934; McMaster University Research Ethics Board approval number 10948; Holland Bloorview Rehabilitation Hospital Research Ethics Board approval number 0086) and all participants provided written informed consent/assent.

**Fig. 1:**
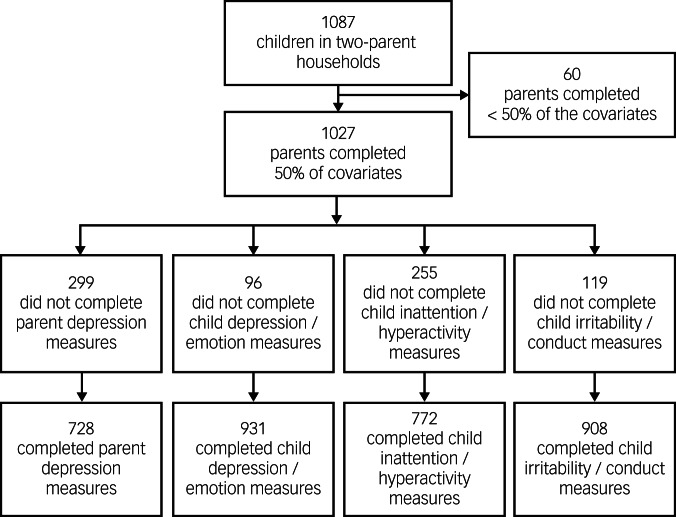
Sample size flow chart.

Parents of children aged 2–18 years (*N* = 1027) completed online questionnaires via REDCap version 13.5.4 for Windows (Vanderbilt University, Nashville, TN, USA; see https://projectredcap.org/) .^31^ To be included in these analyses, participants had to report living in a two-parent household. All data collection was completed from 15 April 2020 to 13 July 2022. For the parent depression outcome, response rates were 67.0% (Fig. 1). For child mental health outcomes, response rate varied by outcome: 85.6% for depression/emotion problems, 71.0% for hyperactivity/inattention and 83.5% for irritability/conduct problems (Fig. 1). To reduce participant burden, particularly for parents with older children whose mental health measures included more questions, not all questions were asked at every data collection point. Analysis was completed up to 31 August 2022.

### Measures

#### Demographics

One parent from each family reported on household income, parent age, parent ethnicity/ancestry, parent role, number of people in the household, dwelling type (house, condominium, apartment), full-time employment of both parents, child age, child sex assigned at birth, child ethnicity/ancestry and child pre-COVID-19 psychiatric diagnoses, using items adapted from the Coronavirus Health Impact Survey (CRISIS) questionnaire (an instrument designed through international collaboration on mental health during the COVID-19 pandemic)^32^ and the CRISIS – Adapted for Autism and Neurodevelopmental Conditions (CRISIS-AFAR).^33^ We did not collect information on the exact date of child pre-COVID-19 psychiatric diagnoses, only that they had been made before the onset of pandemic restrictions in Canada.

#### Structured parenting

Structured parenting was assessed with one item drawn from the Parenting Scale^34^ (‘I am the kind of parent that … sets limits on what my child is allowed to do … lets my child do whatever they want’) and one item on routines in parenting constructed for the purposes of the current study (‘Throughout the day, I provide my child with … a clear and orderly routine … unstructured free time’). Items were measured on a seven-point Likert scale and were averaged. Higher scores indicate higher levels of structured parenting. Parents of children aged 2–5 years provided responses from April 2020 to November 2021 (Cronbach's *α* = 0.76, 95% CI 0.71–0.79). Parents of children aged 6–18 years provided responses from April to July 2020 (Cronbach's *α* = 0.70, 95% CI 0.66–0.77).

#### Shared parenting

Shared parenting was assessed with two items drawn from the Childbearing Attitudes Questionnaire^13^ (‘I am bothered by my partner's lack of involvement in the daily care of the child’; ‘Currently, is the sharing of household tasks a source of tension or conflict between you and your partner?’). Items are measured on a seven-point Likert scale and were averaged. Higher scores indicate higher levels of shared parenting. Parents of children aged 2–5 years provided responses from June 2020 to November 2021 (Cronbach's *α* = 0.76, 95% CI 0.72–0.79). Parents of children aged 6–18 years provided responses from April to July 2020 (Cronbach's *α* = 0.73, 95% CI 0.68–0.78). Structured and shared parenting were very weakly correlated (*r* = 0.04).

#### Parent depression symptoms

Parental depression symptoms were assessed with the Patient Health Questionnaire-8 (PHQ-8), comprising eight items measured with a four-point Likert scale.^35^ Responses were summed and higher scores indicate higher levels of depression symptoms. Parents provided repeated responses (median: 4; range: 1–11) on the PHQ-8 from April 2020 to May 2022.

#### Child depression symptoms

For children ages 2–5 years, parents completed the Strengths and Difficulties Questionnaire (SDQ) Emotional Subscale – Parent Report, comprising five items measured with a three-point Likert scale.^36,37^ For children ages 6–18 years, parents completed the Revised Children's Anxiety and Depression Scale (RCADS-P), a ten-item depression subscale measured with a four-point Likert scale.^38^ Responses were summed for the SDQ Emotional Subscale and *T*-scores were created for RCADS-P. Higher scores indicate higher levels of symptoms. Parents responded repeatedly (median: 4; range: 1–14) from May 2020 to July 2022.

#### Child hyperactivity/inattention symptoms

In children aged 2–5 years, child hyperactivity was measured with the SDQ Hyperactivity Subscale – Parent Report, a five-item subscale measured with a three-point Likert scale.^36,37^ In children aged 6–18 years, hyperactivity and inattention was assessed with the Strengths and Weaknesses of Attention-Deficit/Hyperactivity Disorder Symptoms and Normal Behavior Scale – Parent Report (SWAN), comprising 18 items measured with a seven-point Likert scale.^39^ Sum scores were reversed, with higher scores indicating higher levels of symptoms. Parents responded repeatedly (median: 5; range: 1–14) from July 2020 to July 2022.

#### Child irritability symptoms

For children aged 2–5 years, irritability symptoms were assessed with the SDQ Conduct Problems Subscale – Parent Report, comprising five items measured with a three-point Likert scale.^36,37^ For children aged 6–18 years, irritability was measured with The Irritability and Dysregulation of Emotion Scale, Brief – Parent Report (TIDES), comprising six items measured with a seven-point Likert scale.^40^ Scores were summed, with higher scores indicating higher levels of symptoms. Parents responded repeatedly (median: 4; range: 1–14) from May 2020 to July 2022.

### Data analysis

Data were analysed in R version 4.1.2 for Windows (R Foundation for Statistical Computing, Vienna, Austria; see https://www.R-project.org/) and RStudio version 1.2.1335 for Windows (RStudio Team, PBC, Boston, MA, USA; see http://www.rstudio.com/). As described above, data were collected at different times, using different developmentally appropriate measures. To account for differences in both data collection and in parenting behaviours across child development, we stratified the sample by child age group: 2–5 years old, 6–9 years old, 10–12 years old and 13–18 years old. These age groups were chosen *a priori* based on children's developmental stages requiring more or less explicit limit-setting and more or less instrumental caregiving. We used different pre-processing and data analysis for the age groups 2–5 years and 6–18 years, because of different frequency of exposure assessments. Data were analysed with mixed-effects linear models. Mixed-effects models allow for modelling of longitudinal data, and can handle uneven spacing of repeated measurements and variable numbers of repeated measures per participant/family. Reported results are the estimated fixed effects for the exposure of interest (e.g. structured parenting, shared parenting), after adjusting for the longitudinal nature of the data through fixed effects for changes in date, and random effects to account for correlations within family and participant.

#### Parents and children aged 2–5 years

We used the *mice* package for Windows (van Buuren & Groothuis-Oudshoom; see https://cran.r-project.org/web/packages/mice/index.html) for hierarchical imputation (*n* = 15) of missing item-level covariate and predictor data across multiple assessments (<50% missing data on all items required for analysis).^41–43^ As both the PHQ-8 and the SDQ rely on sum scores, participants had to complete all items on the outcome measure to prevent downward bias.

We analysed associations between repeated assessments of structured and shared parenting (data collection from April 2020 to November 2021) with repeated assessments of depression symptoms (data collection from April 2020 to July 2022), using linear mixed models (*lme4* package for Windows (Bates et al; see https://cran.r-project.org/web/packages/lme4/index.html)).^44^ First, we ran unadjusted models. To account for confounds, we analysed data in adjusted models. In the adjusted parent depression model, we included household income and parent ethnicity/ancestry as potential confounds. In adjusted child mental health models, child ethnicity/ancestry, child sex assigned at birth and PHQ-8 scores were included.

#### Parents and children aged 6–18 years

We used single-level imputation (*n* = 15; *mice*) for missing item-level covariate and predictor data (single assessments), if participants had <50% missing data on all items required for analysis.^41–43^ As the PHQ-8, SWAN and TIDES scales rely on sum scores, no outcomes were imputed to prevent downward bias.

We analysed associations between a single assessment of structured and shared parenting at the start of the COVID-19 pandemic (data collected once from April to July 2020) with repeated measures of parent depression symptoms (data collected from April 2020 through July 2022), using linear mixed models^44^ with the *lme4* package for each age group separately. To account for confounds, we analysed data in adjusted models. In adjusted parent depression models, we included household income, parent role, parent ethnicity/ancestry and child pre-COVID-19 pandemic mental health diagnosis as confounds. These variables have been previously associated with parent mental health and with structured and shared parenting. In adjusted child mental health models, we included household income, child sex assigned at birth, child ethnicity/ancestry, child pre-COVID-19 pandemic mental health diagnosis and PHQ-8 at baseline as confounds. SWAN questions were not asked at baseline. To account for baseline levels of inattention/hyperactivity in the adjusted model, two items measuring hyperactivity and inattention from the CRISIS questionnaire^32^ were included.

## Results

### Participant characteristics

Participant characteristics are shown in Table 1. Because of low variance on specific variables (parent role, household income, previous mental health diagnosis), planned covariates were not included in some adjusted models in children aged 2–5 years.^45^ In contrast, most parents with children aged 6–18 years reported a pre-COVID-19 mental health diagnosis for their child. Therefore, we conducted sensitivity analyses to examine whether results depended on pre-COVID-19 child mental health diagnosis in this age group (Supplementary Material).

**Table 1:** Participant characteristics

Characteristic	% (*n*)	
	Parents with children aged 2–5 years (*n* = 361)	Parents with children aged 6–18 years (*n* = 666)
Parent age (in years), mean (s.d.)	−	43.51 (5.64)
Missing	−	54.50 (363)
Parent role		
Mother	95.57 (345)	79.42 (529)
Father	4.16 (15)	9.91 (66)
Missing	0.28 (1)	10.66 (71)
Child age (in years), mean (s.d.)	4.06 (1.07)	10.48 (3.26)
Missing	0 (0)	10.66 (71)
Child sex assigned at birth		
Male	49.58 (179)	51.5 (343)
Female	50.41 (182)	48.50 (323)
Missing	0 (0)	0 (0)
Ethnicity/ancestry of the parent		
European	62.05 (224)	33.93 (226)
Non-European	22.71 (82)	12.01 (80)
Multiple ethnicities	8.59 (31)	6.15 (41)
Missing	6.65 (24)	47.90 (319)
Ethnicity/ancestry of the child		
European	55.68 (201)	56.91 (379)
Non-European	15.24 (55)	17.72 (118)
Multiple ethnicities	24.38 (88)	24.02 (160)
Missing	4.71 (17)	1.35 (9)
Family income (CAD$)		
<$80 000 per year	14.22 (52)	19.82 (132)
≥$80 000 per year	84.40 (304)	70.12 (467)
Missing	1.39 (5)	10.06 (67)
Dwelling type		
House	76.18 (275)	84.23 (561)
Apartment	18.00 (65)	8.41 (56)
Condominium	−	6.31 (42)
Missing	5.82 (21)	1.05 (7)
Number of people in the household, mode (range)	−	4 (9)
Missing	−	0 (0)
Employment type		
Responding parent (full time)	30.19 (109)	59.15 (394)
Missing	15.51 (56)	1.05 (7)
Partner parent (full time)	82.83 (299)	82.73 (551)
Missing	11.08 (40)	0 (0)
Previous mental health diagnosis of child		
Yes	2.77 (10)	57.05 (380)
No	96.12 (347)	42.94 (286)
Missing	1.11 (4)	0 (0)
Structured parenting, mean (s.d.)^a,b^	4.52 (1.19)	4.51 (1.21)
Missing	1.5 (n)	11.6 (77)
Satisfaction with shared parenting, mean (s.d.)^a^^,^^b^	5.02 (1.65)	4.72 (1.75)
Missing	7.6 (n)	14.26 (95)
PHQ-8, mean (s.d.)^a,c^	4.83 (4.75)	7.34 (5.43)
Missing	0 (0)	11.56 (77)
SDQ Emotional Subscale scores across all measures^c^	1.76 (1.85)	−
Missing	0 (0)	−
RCADS-P depression subscale at baseline, mean (s.d.)^d^	−	60.26 (16.84)
Missing	−	4.80 (32)
SDQ Hyperactivity Subscale scores across all measures^c^	4.83 (4.75)	−
Missing	0 (0)	−
Hyperactivity/inattention at baseline, mean (s.d.)^e^	−	6.05 (2.05)
missing	−	8.71 (58)
SDQ Conduct Problems Subscale problem scores across all measures^f^	1.65 (1.52)	−
Missing	0 (0)	−
TIDES at baseline, mean (s.d.)^g^	−	1.27 (8.77)
Missing	−	8.11 (54)

PHQ-8, Patient Health Questionnaire-8; SDQ, Strengths and Difficulties Questionnaire; RCADS-P, Revised Children's Anxiety and Depression Scale; TIDES, The Irritability and Dysregulation of Emotion Scale, Brief; n, number of participants.

a. For parents with children aged 2–5 years, results reflect an average across all measurements; for parents with children aged 6–18 years, results reflect an average of the baseline (only) measure.

b. Range: 1–7.

c. Range: 0–10.

d. Range: 35.16–126.08.

e. Range: 2–10.

f. Range: 0–8.

g. Range: −18 to 18.

### Parent depression symptoms

**Fig. 2:**
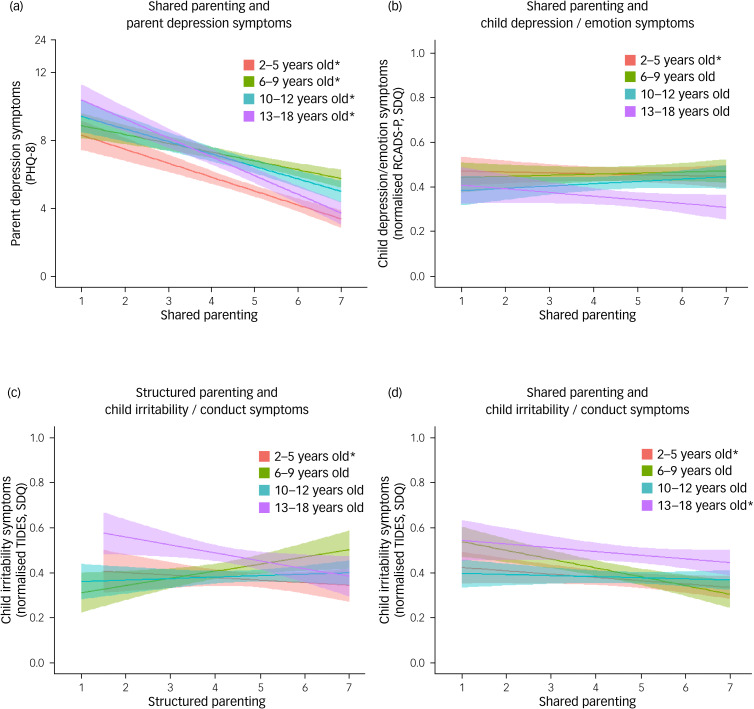
(a) Shared parenting and parent symptoms of depression. (b) Shared parenting and child symptoms of depression/emotional problems. (c) Structured parenting and child symptoms of irritability/conduct problems. (d) Shared parenting and child symptoms of irritability/conduct problems. All analyses stratified by child developmental groups: 2–5 years, 6–9 years, 10–12 years and 13–18 years. Statistically significant results are indicated by an asterisk (*) in the key.

Structured parenting was not associated with parent depression symptoms across the pandemic in any of the child age groups (Table 2). However, higher levels of shared parenting were associated with lower parent depression symptoms (Table 2, Fig. 2(a)).

**Table 2:** Association between structured and shared parenting and mental health

Domain and covariates	Unadjusted		Adjusted^a^	
	*β* (95% CI)	*P*-value	*β* (95% CI)	*P*-value
Parent depression^a^				
Age 2–5 years				
Structured parenting	−0.040 (−0.093 to 0.014)	0.150	−0.034 (−0.091 to 0.023)	0.240
Shared parenting	−0.100 (−0.164 to −0.035)	**0.003**	−0.093 (−0.160 to −0.025)	**0.008**
Age 6–9 years				
Structured parenting	−0.085 (−0.190 to 0.020)	0.112	−0.083 (−0.184 to 0.019)	0.109
Shared parenting	−0.190 (−0.298 to −0.083)	**0.001**	−0.165 (−0.271 to −0.060)	**<0.001**
Age 10–12 years				
Structured parenting	−0.069 (−0.204 to 0.067)	0.329	−0.081 (−0.217 to 0.055)	0.241
Shared parenting	−0.172 (−0.295 to −0.049)	**0.006**	−0.166 (−0.287 to −0.045)	**0.007**
Age 13–18 years				
Structured parenting	−0.041 (−0.183 to 0.141)	0.572	−0.049 (−0.190 to 0.091)	0.490
Shared parenting	−0.323 (−0.459 to −0.189)	**<0.001**	−0.317 (−0.452 to −0.182)	**<0.001**
Child depression^b^				
Age 2–5 years (SDQ Emotional Subscale)				
Structured parenting	−0.002 (−0.050 to 0.046)	0.931	0.001 (−0.047 to 0.049)	0.956
Shared parenting	−0.072 (−0.125 to −0.020)	**0.007**	−0.065 (−0.120 to −0.011)	**0.019**
Age 6–9 years (RCADS-P)				
Structured parenting	−0.057 (−0.160 to 0.045)	0.271	−0.043 (−0.132 to 0.046)	0.343
Shared parenting	−0.173 (−0.275 to −0.071)	**<0.001**	−0.070 (−0.163 to 0.024)	0.143
Age 10–12 years (RCADS-P)				
Structured parenting	−0.075 (−0.205 to 0.055)	0.260	−0.037 (−0.154 to 0.080)	0.534
Shared parenting	−0.124 (−0.248 to 0.001)	0.053	−0.066 (−0.180 to 0.049)	0.261
Age 13–18 years (RCADS-P)				
Structured parenting	−0.168 (−0.299 to −0.036)	**0.013**	−0.110 (−0.224 to 0.004)	0.060
Shared parenting	−0.218 (−0.344 to −0.092)	**<0.001**	−0.109 (−0.226 to 0.008)	0.068
Child hyperactivity and inattention^b^				
Age 2–5 years (SDQ Hyperactivity Subscale)				
Structured parenting	−0.043 (−0.091 to 0.005)	0.077	−0.0419 (−0.090 to 0.005)	0.084
Shared parenting	−0.051 (−0.108 to 0.006)	0.077	−0.0442 (−0.100 to 0.012)	0.121
Age 6–9 years (SWAN)				
Structured parenting	0.002 (−0.115 to 0.117)	0.977	0.007 (−0.080 to 0.093)	0.879
Shared parenting	−0.092 (−0.213 to 0.029)	0.135	−0.010 (−0.104 to 0.083)	0.829
Age 10–12 years (SWAN)				
Structured parenting	0.036 (−0.128 to 0.199)	0.668	0.071 (−0.054 to 0.195)	0.265
Shared parenting	−0.050 (−0.203 to 0.103)	0.514	0.047 (−0.074 to 0.167)	0.443
Age 13–18 years (SWAN)				
Structured parenting	0.049 (−0.125 to 0.222)	0.583	−0.013 (−0.168 to 0.143)	0.873
Shared parenting	−0.100 (−0.260 to 0.061)	0.223	−0.084 (−0.238 to 0.071)	0.287
Child irritability^b^				
Age 2–5 years (SDQ Conduct Problems Subscale)				
Structured parenting	−0.056 (−0.108 to −0.003)	**0.038**	−0.054 (−0.107 to −0.001)	**0.047**
Shared parenting	−0.091 (−0.149 to −0.032)	**0.003**	−0.086 (−0.147 to −0.025)	**0.006**
Age 6–9 years (TIDES)				
Structured parenting	0.027 (−0.083 to 0.137)	0.629	0.037 (−0.067 to 0.141)	0.480
Shared parenting	−0.134 (−0.242 to −0.026)	**0.015**	−0.063 (−0.168 to 0.043)	0.243
Age 10–12 years (TIDES)				
Structured parenting	−0.010 (−0.145 to 0.124)	0.882	0.032 (−0.094 to 0.157)	0.620
Shared parenting	−0.133 (−0.258 to −0.007)	**0.038**	−0.096 (−0.213 to 0.021)	0.107
Age 13–18 years (TIDES)				
Structured parenting	0.019 (−0.115 to 0.153)	0.780	0.012 (−0.115 to 0.139)	0.850
Shared parenting	−0.302 (−0.430 to −0.174)	**<0.001**	−0.270 (−0.399 to −0.140)	**<0.001**

Bold values were statistically significant. To account for testing three different outcomes (emotional problems, hyperactivity, conduct problem) for children aged 2–5 years, applying the Bonferroni correction (0.05/3), results in a critical value for statistical significance of 0.017; and to account for three different outcomes (depression symptoms, hyperactivity/inattention symptoms, irritability symptoms) for children aged 6–18 years, applying the Bonferroni correction (0.05/3), results in a critical value for statistical significance of 0.017. SDQ, Strengths and Difficulties Questionnaire; RCADS-P, Revised Children's Anxiety and Depression Scale; SWAN, Strengths and Weaknesses of Attention-Deficit/Hyperactivity Disorder Symptoms and Normal Behavior Scale – Parent Report; TIDES, The Irritability and Dysregulation of Emotion Scale, Brief.

a. The model with parents with children aged 2–5 years was adjusted for income and parent ethnicity; models with parents with children aged 6–18 years were adjusted for income, parent ethnicity, responding parent role and previous mental health diagnosis for child.

b. The model with children aged 2–5 years was adjusted for child gender assigned at birth, child ethnicity and parent depression; models with children aged 6–18 years were adjusted for income, child gender assigned at birth, child ethnicity, previous mental health diagnosis for child and parent depression

### Child depression symptoms

Structured parenting was not associated with child depression/emotion problems (Table 2). However, in children aged 2–5 years, higher levels of shared parenting were associated with lower levels of child emotional problems, but not depression, in other developmental groups (Table 2, Fig. 2(b)).

### Child hyperactivity and inattention symptoms

Neither structured nor shared parenting were significantly associated with child hyperactivity or inattention/hyperactivity symptoms (Table 2).

### Child irritability symptoms

Among children aged 2–5 years, higher levels of structured parenting and higher levels of shared parenting across the study were significantly associated with lower child conduct problems (Table 2, Figs. 2(b), 2(c)). We did not detect significant associations between structured parenting and child irritability in other developmental groups (Table 2, Fig. 2(c)). However, among children aged 13–18 years, higher levels of shared parenting at the start of the COVID-19 pandemic were associated with lower irritability symptoms across the study (Table 2, Fig. 2(d)).

## Discussion

The objectives of this study were to examine the association of structured and shared parenting with parent and child mental health. Our findings suggest that higher levels of shared parenting are associated with fewer parent depression symptoms in parents with children in all age groups (2–5, 6–9, 10–12 and 13–18 years of age), but structured parenting was not associated with parent symptoms of depression in parents with children in any age group. Among children aged 2–5 years, higher levels of shared parenting were associated with fewer child emotion and conduct problems, but were not associated with hyperactivity symptoms. Among children aged 2–5 years, higher levels of structured parenting were associated with fewer child conduct problems, but were not associated with emotion problems or hyperactivity symptoms. Among children aged 6–9 years, neither shared parenting nor structured parenting were associated with depression, irritability or inattention/hyperactivity symptoms. Among children aged 10–12 years, neither shared parenting nor structured parenting were associated with depression, irritability or inattention/hyperactivity symptoms. Among adolescents aged 13–18 years, shared parenting was associated with less irritability, but was not associated with depression or inattention/hyperactivity symptoms. Among adolescents (aged 13–18 years), structured parenting was not associated with depression, irritability or inattention/hyperactivity symptoms.

### Structured parenting

During the COVID-19 pandemic, externally supported structure was often absent.^1^ This loss placed the burden of creating and maintaining structure on parents, such as keeping up with school-related tasks, sufficient physical activities and cognitive stimulation, and play. Pandemic-related lockdowns have been associated with reduced structured parenting.^1,46,47^ Our findings on structured parenting and parent depression contrast studies conducted before the COVID-19 pandemic, which found that higher levels of structured parenting were associated with better well-being in mothers^10^ and decreased depression in parents.^8^ In contrast to a cross-sectional, COVID-19-pandemic-related study showing an association between structured parenting and parent depression,^46^ our longitudinal results did not show any such association. Moreover, despite cross-sectional, COVID-19-pandemic-related associations between structured parenting and child mental health,^47^ we did not find much support for this association across domains and age groups. Structured parenting was only associated with decreased conduct problems in children aged 2–5 years, which is in agreement with previous literature.^47^

Structured parenting was not associated with parent mental well-being and, with the exception of conduct problems in children aged 2–5 years, was not associated with child mental health in any other age group or domain. This might be because parents prioritised other factors during this chaotic context rather than structuring the family environment. Given the number of concurrent changes to family life and the absence of externally supported structure, the consistent and effective implementation of structured parenting may have been more than parents were able or willing to take on, and thus may not have affected mental health. Other factors may have contributed more to parent and child mental health, such as lockdown policy stringency,^48^ COVID-19-related stress,^49^ material deprivation^49^ or pre-existing child mental health diagnosis (see Supplementary Table S2). A core component of structured parenting is consistency, which would have been particularly challenging considering the frequent and short-notice changes to public health measures (Supplementary Fig. 1). Our findings support future investigations on the use and limits of structured parenting under the chaotic and inflexible life conditions that some families experience outside of the context of the COVID-19 pandemic.

### Shared parenting

Shared parenting was associated with better parent mental health across all age groups and better child mental health (ages 2–5 years: emotion and conduct problems; ages 13–18 years: irritability). Sharing of household and childcare labour is a highly discussed topic, in research as well as in the general public.^50^ Studies from before the COVID-19 pandemic indicate that women spend two to ten times more time on unpaid household and childcare labour than men, across all levels of income and all locations studied to date.^51^ Even when fathers worked less and made less money than their female partners, they engaged in less housework on average.^52^ Before the COVID-19 pandemic, paternal involvement in childcare was associated with their own gender-role views, and not those of their female partner.^53^ However, during the pandemic, a different pattern emerged. For instance, a programme in Germany, intended to buffer economic and labour market problems, allowed employees to retain their jobs and salary but work reduced hours during lockdown phases. Fathers who participated in this programme engaged in more housework and childcare, particularly fathers with low or medium educational level.^54^ In the USA, parents who reported having no help in household and childcare labour were more likely to reduce paid work hours or completely drop out of the paid workforce. Thus, shared parenting may support workforce participation, with economic benefit for the family.^55^

Changes to the macrosystem in the COVID-19 pandemic context required parents to balance the demands of paid work (or the concerns of lost work), instrumental and affective care of children, negotiating/managing online school and other family demands in a time of great uncertainty. During the COVID-19 pandemic, both parents and children spent more time in the home, resulting in increased household labour and childcare responsibilities for parents. With a few exceptions (e.g. Sevilla and Smith^56^), multiple reports indicate more equitable sharing of household and childcare labour by parents in diverse cultures during the COVID-19 pandemic.^54,55,57–59^ When the division of household labour and childcare was perceived to be more equitable, mothers reported fewer relationship problems^60^ and better coping skills.^61^

Perhaps counterintuitively, we found that shared parenting had the largest effect on the mental health of parents with adolescent children compared with parents with younger children. This may reflect the different demands of parenting adolescents in particular.^17^ Likewise, we also found higher levels of shared parenting were associated with decreased symptoms of irritability in adolescents. Positive family dynamics (i.e. shared parenting) may have buffered the effects of social isolation for adolescents during the pandemic.^62^ Adolescents who reported their parents had poor marital quality had higher rates of mental health problems as adults.^63^ Parenting conflicts, particularly those related to co-parenting, have also been associated longitudinally with worse parent–adolescent relationship quality,^16,63^ and family conflict is the leading cause of mental distress in adolescents.^64^

We found that higher levels of shared parenting were associated with fewer emotional and conduct problems in children aged 2–5 years. We did not find any studies specifically examining the role of shared parenting in child mental health during the pandemic. However, a 2010 meta-analysis reported small effects between both parent cooperation and conflict and child externalising symptoms, with larger effect sizes within clinical populations.^65^ In our sensitivity analyses, effect sizes for shared parenting were larger for parent and child depression symptoms when the child had a pre-existing mental health diagnosis compared with those who did not (Supplementary Tables 7 and 8). Similarly, there were small effects between both parent cooperation and conflict and child internalising symptoms, with larger effect sizes among younger children.^65^ Although household labour and childcare have become more equitably shared between parents during the COVID-19 pandemic,^54,55,57–59^ it is unclear whether this adjustment will precipitate more durable changes in the division of household and childcare labour. This might benefit a broad spectrum of outcomes,^4,13–16,55^ including the current findings on parent and child mental health.

Shared parenting is amenable to intervention. Two recent systematic reviews and meta-analysis identified positive effects of co-parenting interventions on parent well-being^6,66^ for at-risk families and families with no known risks.^6^ Further, co-parenting interventions can be effective in non-traditional or multigenerational families.^66^ Co-parenting interventions fostering shared parenting, even in times of crisis,^67^ might be beneficial for parents, children and labour participation.^4,13–16,55^

### Limitations and strengths

An important limitation in this study is our sample restriction to include only two-parent households. Future work should consider diverse family structures (e.g. single parents, multigenerational households) and diverse parents (e.g. LGBTQI+). This study was also limited by convenience sampling, which may not be representative of the general population in socioeconomic dimensions, and source bias, as all measures in this study were reported by parents. Understanding how different families and parents experiencing different contextual stressors may enact structured and shared parenting practices, and how these may buffer their own mental health and their child's mental health, will be important for clinical translation of these findings.

Despite these limitations, our study has several strengths, including longitudinal design in a large sample, enriched participation of parents whose children have a pre-existing mental health or neurodevelopmental disorder, and a powerful statistical approach making use of all available data. Our study is the first to our knowledge that examines the role of both structured and shared parenting in the same model. Further, we examine both parent and child mental health across the full span of child development, consider multiple mental health outcomes in children, and test how these family management systems affect children and adolescents with and without pre-existing mental health diagnoses in the same study.

In conclusion, shared parenting contributes meaningfully to parent and child mental health in two-parent households, even under times of stress. Shared parenting may be a target for intervention among parents and children experiencing mental health difficulties. Support of shared parenting responsibilities (parental leave policies guaranteeing paternal leave, flexible work-from-home policies, public health engagement, clinical interventions) may be a promising strategy to ameliorate parent and child mental health during the COVID-19 pandemic recovery and beyond.

## Supporting information

Cost et al. supplementary material Cost et al. supplementary material

## Data Availability

The data that support these findings are not publicly available because the data contain information that could compromise the privacy of the research participants.
