# Supplementary material for: The role of parenting practices in parent and child mental health over time
Source: BJPsych Open. 2023 Aug 8;9(5):e147. doi: 10.1192/bjo.2023.529 (PMC10594096; doi:10.1192/bjo.2023.529)
Supplement: Cost et al. supplementary material [file S205647242300529Xsup001.docx]

**Supplement**

**Supplement Figure 1.** Timeline of COVID-19 pandemic related context over the course of the study. Grey boxes indicate lockdown phases (April-June 2020; December 2020- May 2021; November 2021-Mach 2022); blue boxes represent opening phases with some restrictions (July-August 2020; September-November 2020; June-October 2021); green boxes indicate the transition phase wherein restrictions on in-person learning and recreation facilities were eased, mask mandates were lifted, and vaccines were available for participants across all age bands in this study (April-June 2022; July 2022).

*Table S1:* Association between Parent Depression and Shared and Structured Parenting across the pandemic. (Parents with children 2-5 years)- Adjusted Model

| Covariates and Confounders | β (95% CI) | P value |
| --- | --- | --- |
| Time | **0.0006 (0.0002 to 0.0010)** | **0.0023*** |
| Structured parenting | -0.0343(-0.0913 to0.0227) | 0.2384 |
| Shared parenting | **-0.0925(-0.1603 to -0.0247)** | **0.0076*** |
| Income (>80000) | -0.0484(-0.4670 to 0.3702) | 0.8189 |
| Ethnicity (Non-European) | 0.0856(-0.2224 to 0.3936) | 0.5855 |
| Ethnicity (Multiple ethnicities) | -0.0993(-0.5603 to 0.3616) | 0.6724 |

*Table S2:* Association between Parent Depression across the pandemic and Shared and Structured Parenting at the beginning of the pandemic. (Parents with children 6-18 years)- Adjusted Model

|  | Parents with children 6-9 years | | Parents with children 10-12 years | | Parents with children 13-18 years | |
| --- | --- | --- | --- | --- | --- | --- |
|  | *β (95% CI)* | *P value* | *β (95% CI)* | *P value* | *β (95% CI)* | *P value* |
| Time | **-0.0002(-0.0004 to -0.0001)** | **0.007*** | -0.0001(-0.0004 to 0.0001) | 0.305 | -0.0001(-0.0004 to 0.0001) | 0.291 |
| Structured parenting | -0.0830 (-0.1847 to 0.0186) | 0.109 | -0.0811(-0.2167 to 0.0545) | 0.241 | -0.0494(-0.1900  to 0.0912) | 0.490 |
| Shared parenting | **-0.1652(-0.2708 to-0.0597)** | **0.002*** | **-0.1664(-0.2874 to -0.0454)** | **0.007*** | **-0.3170(-0.4520**  **to -0.1820)** | **<0.0001*** |
| Income  ( >80000) | -0.1920(-0.4231 to 0.0391) | 0.103 | -0.1441(-0.4340 to 0.1457) | 0.329 | -0.1061(-0.4407  to 0.2285) | 0.531 |
| Previous MH diagnosis of child | **0.3549(0.1434 to 0.5663)** | **0.001*** | **0.3143(0.0413 to 0.5873)** | **0.024*** | ***0.3050(-0.0432***  ***to 0.6532)*** | ***0.086*** |
| Ethnicity (Non-European) | 0.0579(-0.2026 to 0.3185) | 0.662 | -0.2381(-0.5999 to 0.1237) | 0.194 | 0.0568(-0.3150  to 0.4287) | 0.762 |
| Ethnicity (Multiple ethnicities) | 0.1001(-0.2245 to 0.4246) | 0.540 | -0.1147(-0.5892 to 0.3599) | 0.631 | -0.0216(-0.3210  to 0.2778) | 0.887 |
| Parent role (Father) | ***-0.3070(-0.6173 to 0.0032)*** | ***0.052*** | -0.0759(-0.4709 to 0.3192) | 0.706 | -0.4853(-1.0781  to 0.1076) | 0.108 |

*Table S3:* Association between Child Emotional Problems, Hyperactivity, Conduct problems and Shared and Structured Parenting across the pandemic. (Parents with children 2-5 years)- Adjusted Model

|  | Emotional | | Hyperactivity | | Conduct | |
| --- | --- | --- | --- | --- | --- | --- |
|  | *β (95% CI)* | *P value* | *β (95% CI)* | *P value* | *β (95% CI)* | *P value* |
| Time | ***0.0002(0.000 to 0.0004)*** | ***0.078*** | 0.0001(-0.0001 to 0.0003) | 0.436 | **-0.0004(-0.0006 to -0.0002)** | **0.001*** |
| Structured parenting | 0.001(-0.047 to 0.049) | 0.956 | ***-0.042(-0.090 to 0.006)*** | ***0.084*** | **-0.054(-0.107 to -0.001)** | **0.047*** |
| Shared parenting | **-0.065(-0.120 to -0.011)** | **0.019*** | -0.044(-0.100 to 0.012) | 0.121 | **-0.086(-0.147 to -0.025)** | **0.006*** |
| Ethnicity (Non-European) | -0.055(-0.354 to 0.245) | 0.719 | -0.020(-0.290 to 0.251) | 0.887 | 0.107(-0.247 to 0.460) | 0.550 |
| Ethnicity (Multiple ethnicities) | 0.049(-0.188 to 0.287) | 0.681 | -0.068(-0.271 to 0.135) | 0.511 | -0.045(-0.283 to 0.194) | 0.710 |
| Child Sex (Female) | -0.019(-0.219 to 0.182) | 0.856 | **-0.337(-0.533 to -0.142)** | **0.001*** | **-0.204(-0.403 to -0.004)** | **0.046*** |
| Parent PHQ-8 | **0.089(0.016 to 0.163)** | **0.018*** | 0.073(0.014 to 0.132) | 0.016 | 0.057(-0.017 to 0.131) | 0.126 |

*Table S4:* Association between Child Depression across the pandemic and Shared and Structured Parenting at the beginning of the pandemic. (Parents with children 6-18 years)- Adjusted Model

|  | Children 6-9 years | | Children 10-12 years | | Children 13-18 years | |
| --- | --- | --- | --- | --- | --- | --- |
|  | β (95% CI) | P value | β (95% CI) | P value | β (95% CI) | P value |
| Time | <0.000(-0.0001 to 0.0001) | 0.729 | <0.000(-0.0001 to 0.0002) | 0.627 | <0.000(-0.0001 to  0.0002) | 0.545 |
| Structured parenting | -0.0431(-0.132 to 0.046) | 0.343 | -0.037(-0.154 to  0.080) | 0.534 | ***-0.110(-0.224 to***  ***0.004)*** | ***0.060*** |
| Shared parenting | -0.0697(-0.163 to  0.024) | 0.143 | -0.066(-0.180 to  0.049) | 0.261 | ***-0.109(-0.226 to***  ***0.008)*** | ***0.068*** |
| Income  (> 80,000CAD) | -0.0367(-0.222 to  0.149) | 0.697 | -0.166(-0.456 to  0.125) | 0.259 | -0.060(-0.361 to  0.240) | 0.693 |
| Previous MH dx of child | **0.5557(0.381 to**  **0.730)** | **0.000*** | **0.637(0.405 to**  **0.868)** | **0.000*** | **0.728(0.433 to**  **1.022)** | **0.000*** |
| Ethnicity (non-European) | -0.0482(-0.279 to  0.182) | 0.681 | -0.026(-0.334 to  0.282) | 0.868 | -0.036(-0.376 to  0.305) | 0.838 |
| Ethnicity (Multiple) | -0.0022(-0.211 to  0.206) | 0.983 | 0.058(-0.206 to  0.323) | 0.667 | -0.006(-0.274 to  0.261) | 0.963 |
| Child sex (female) | -0.0634(-0.234 to  0.107) | 0.464 | -0.051(-0.275 to  0.173) | 0.656 | **0.522(0.293 to**  **0.750)** | **0.000*** |
| Parent PHQ-8 | **0.2824(0.192 to**  **0.373)** | **0.000*** | **0.257(0.135 to**  **0.379)** | **0.000*** | **0.272(0.152 to 0.392)** | **0.000*** |

*Table S5:* Association between Child Hyperactivity/Inattention across the pandemic and Shared and Structured Parenting at the beginning of the pandemic. (Parents with children 6-18 years)- Adjusted Model

|  | Children 6-9 years | | Children 10-12 years | | Children 13-18 years | |
| --- | --- | --- | --- | --- | --- | --- |
|  | *β (95% CI)* | *P value* | *β (95% CI)* | *P value* | *β (95% CI)* | *P value* |
| Time | **<0.000(**  **-0.000 to -0.0001)** | **0.009*** | **<0.000** | **0.025*** | <0.000(<0.000 to 0.0002) | 0.477 |
| Structured parenting | 0.007(-0.080 to  0.093) | 0.879 | 0.071(-0.054 to  0.195) | 0.265 | -0.013(-0.168 to  0.143) | 0.873 |
| Shared parenting | -0.010(-0.104 to  0.083) | 0.829 | 0.047(-0.074 to  0.167) | 0.443 | -0.084(-0.238 to  0.071) | 0.287 |
| Income  (> 80,000CAD) | **0.421(0.320 to 0.522)** | **0.000*** | **0.566(0.427 to**  **0.705)** | **0.000*** | **0.330(0.156 to**  **0.504)** | **0.000*** |
| Previous MH dx of child | -0.127(-0.363 to  0.109) | 0.286 | 0.008(-0.273 to  0.289) | 0.957 | ***-0.319(-0.679 to***  ***0.042)*** | ***0.082*** |
| Baseline Hyperactivity/ Inattention | **0.553(0.361 to**  **0.745)** | **0.000*** | **0.345(0.065 to**  **0.626)** | **0.016*** | ***0.410(-0.019 to***  ***0.839)*** | ***0.061*** |
| Ethnicity (non-European) | 0.101(-0.136 to  0.337) | 0.404 | 0.104(-0.226 to  0.433) | 0.538 | 0.108(-0.382 to  0.597) | 0.665 |
| Ethnicity (Multiple) | 0.060(-0.152 to  0.271) | 0.580 | -0.108(-0.382 to  0.166) | 0.438 | -0.066(-0.438 to  0.307) | 0.728 |
| Child sex (female) | **-0.207(-0.382 to**  **-0.032)** | **0.020*** | ***-0.214(-0.464 to***  ***0.036)*** | ***0.094*** | **-0.569(-0.886 to**  **-0.252)** | **0.000*** |
| Parent PHQ-8 | -0.017(-0.112 to  0.079) | 0.734 | 0.035(-0.086 to  0.156) | 0.568 | -0.036(-0.200 to  0.127) | 0.663 |

*Table S6:* Association between Child Irritability across the pandemic and Shared and Structured Parenting at the beginning of the pandemic. (Parents with children 6-18 years)- Adjusted Model

|  | Children 6-9 years | | Children 10-12 years | | Children 13-18 years | |
| --- | --- | --- | --- | --- | --- | --- |
|  | *β (95% CI)* | *P value* | *β (95% CI)* | *P value* | *β (95% CI)* | *P value* |
| Time | **-0.0003(-0.0004 to -0.0001)** | **0.000*** | **<0.000(-0.001**  **To 0.000)** | **0.001*** | 0.000(-0.0002 to 0.0002) | 0.822 |
| Structured parenting | 0.037(-0.067 to  0.141) | 0.480 | 0.032(-0.094 to  0.157) | 0.620 | 0.012(-0.115 to 0.139) | 0.849 |
| Shared parenting | -0.063(-0.168 to  0.043) | 0.243 | -0.096(-0.213 to  0.021) | 0.107 | **-0.270(-0.399 to**  **-0.140)** | **0.000*** |
| Income  (> 80,000CAD) | 0.053(-0.165 to  0.271) | 0.629 | -0.059(-0.338 to  0.221) | 0.678 | 0.130(-0.170 to  0.430) | 0.395 |
| Previous MH dx of child | **0.528(0.320 to**  **0.736)** | **0.000*** | **0.537(0.281 to**  **0.793)** | **0.000*** | **0.620(0.293 to**  **0.947)** | **0.000*** |
| Ethnicity (non-European) | -0.181(-0.478 to  0.115) | 0.230 | -0.331(-0.697 to  0.036) | 0.077 | -0.013(-0.391 to  0.365) | 0.946 |
| Ethnicity (Multiple) | -0.019(-0.268 to  0.230) | 0.882 | -0.014(-0.302 to  0.274) | 0.923 | 0.250(-0.050 to  0.551) | 0.102 |
| Child sex (female) | 0.09(-0.113 to 0.292) | 0.384 | 0.003(-0.243 to  0.248) | 0.983 | -0.165(-0.420 to 0.091) | 0.207 |
| Parent PHQ-8 | **0.168(0.064 to 0.272)** | **0.002*** | **0.164(0.040 to**  **0.288)** | **0.009*** | 0.092(-0.042 to  0.225) | 0.178 |

**Sensitivity Analysis**

*Table S7:* Association between parent depression across the pandemic and Shared and Structured Parenting at the beginning of the pandemic by child pre-existing mental health diagnosis (Parents with children 6-18 years)- Adjusted Model

|  | Parents with children who have no pre-existing mental health conditions | | Parents with children who have pre-existing mental health conditions | |
| --- | --- | --- | --- | --- |
|  | *β (95% CI)* | *P value* | *β (95% CI)* | *P value* |
| Time | **-0.0002(-0.0004 to 0.000)** | **0.0141*** | ***-0.0002(-0.0003 to 0.000)*** | ***0.089*** |
| Structured parenting | -0.0524(-0.152 to 0.047) | 0.3019 | -0.035(-0.132 to 0.061) | 0.474 |
| Shared parenting | ***-0.0950(-0.199 to 0.009)*** | ***0.0734*** | **-0.285(-0.378 to -0.193)** | **0.000*** |
| Income (>80000) | -0.1645(-0.374 to 0.045) | 0.1237 | **-0.221(-0.440 to -0.002)** | **0.048*** |
| Ethnicity (Non-European) | -0.0598(-0.278 to 0.158) | 0.5899 | -0.073(-0.385 to 0.239) | 0.643 |
| Ethnicity  (Multiple ethnicities) | -0.0654-0.444 to 0.313) | 0.7316 | 0.002(-0.235 to 0.240) | 0.984 |
| Parent role (Father) | -0.2231(-0.493 to 0.047) | 0.1053 | -0.318(-0.706 to 0.070) | 0.108 |

*Table S8:* Association between child depression across the pandemic and Shared and Structured Parenting at the beginning of the pandemic by child pre-existing mental health diagnosis (Children aged6-18 years)- Adjusted Model

|  | Children with no pre-existing mental health conditions | | Children with pre-existing mental health conditions | |
| --- | --- | --- | --- | --- |
|  | *β (95% CI)* | *P value* | *β (95% CI)* | *P value* |
| Time | 0.000(-0.0001 to 0.0001) | 0.719 | 0.0009(-0.0001 to 0.0001) | 0.841 |
| Structured parenting | -0.055(-0.137 to 0.027) | 0.186 | -0.053(-0.135 to 0.029) | 0.206 |
| Shared parenting | -0.049(-0.139 to 0.041) | 0.285 | **-0.097(-0.180 to -0.014)** | **0.022*** |
| Income (>80000) | -0.114(-0.290 to 0.063) | 0.207 | -0.055(-0.254 to 0.145) | 0.591 |
| Ethnicity (Non-European) | -0.040(-0.225 to 0.145) | 0.670 | -0.007(-0.287 to 0.273) | 0.959 |
| Ethnicity  (Multiple ethnicities) | 0.084(-0.109 to 0.278) | 0.393 | -0.018(-0.214 to 0.178) | 0.857 |
| Child sex (Female) | 0.044(-0.115 to 0.202) | 0.589 | 0.134(-0.031 to 0.299) | 0.112 |
| Parent PHQ-8 | **0.256(0.171 to 0.340)** | **0.000*** | **0.281(0.192 to 0.371)** | **0.000*** |

*Table S9:* Association between child hyperactivity and inattention across the pandemic and Shared and Structured Parenting at the beginning of the pandemic by child pre-existing mental health diagnosis (Children aged6-18 years)- Adjusted Model

|  | Children with no pre-existing mental health conditions | | Children with pre-existing mental health conditions | |
| --- | --- | --- | --- | --- |
|  | *β (95% CI)* | *P value* | *β (95% CI)* | *P value* |
| Time | **0.000(-0.0004 to 0.000)** | **0.014*** | **0.000(-0.0003 to 0.000)** | **0.032*** |
| Structured parenting | 0.070(-0.029 to 0.168) | 0.164 | -0.010(-0.097 to 0.077) | 0.822 |
| Shared parenting | 0.044(-0.073 to 0.160) | 0.463 | -0.022(-0.107 to 0.064) | 0.621 |
| Hyperactivity/Inattention: baseline | **0.388(0.268 to 0.509)** | **0.000*** | **0.501(0.407 to 0.594)** | **0.000*** |
| Income (>80000) | ***-0.214(-0.470 to 0.041)*** | ***0.099*** | -0.087(-0.307 to 0.133) | 0.431 |
| Ethnicity (Non-European) | -0.075(-0.311 to 0.160) | 0.530 | ***0.273(-0.039 to 0.586)*** | ***0.086*** |
| Ethnicity  (Multiple ethnicities) | -0.097(-0.338 to 0.143) | 0.427 | 0.004(-0.197 to 0.205) | 0.969 |
| Child sex (Female) | ***-0.191(-0.396 to 0.014)*** | ***0.068*** | **-0.378(-0.554 to -0.202)** | **0.000*** |
| Parent PHQ-8 | 0.042(-0.063 to 0.147) | 0.430 | -0.034(-0.124 to 0.056) | 0.462 |

*Table S10:* Association between child irritability across the pandemic and Shared and Structured Parenting at the beginning of the pandemic by child pre-existing mental health diagnosis (Children aged 6-18 years)- Adjusted Model

|  | Children with no pre-existing mental health conditions | | Children with pre-existing mental health conditions | |
| --- | --- | --- | --- | --- |
|  | *β (95% CI)* | *P value* | *β (95% CI)* | *P value* |
| Time | **-0.0002(-0.0004 to -0.0001)** | **0.006*** | **-0.0002(-0.0004 to -0.0001)** | **0.000*** |
| Structured parenting | -0.007(-0.104 to 0.090) | 0.888 | ***0.082(-0.009 to 0.173)*** | ***0.077*** |
| Shared parenting | **-0.135(-0.241 to -0.030)** | **0.012*** | **-0.109(-0.198 to -0.020)** | **0.017*** |
| Income (>80000) | -0.015(-0.248 to 0.219) | 0.902 | 0.045(-0.162 to 0.252) | 0.667 |
| Ethnicity (Non-European) | **-0.252(-0.491 to -0.014)** | **0.038*** | -0.145(-0.482 to 0.192) | 0.397 |
| Ethnicity (Multiple ethnicities) | -0.048(-0.291 to 0.195) | 0.699 | 0.100(-0.114 to 0.314) | 0.359 |
| Child sex (Female) | 0.021(-0.172 to 0.214) | 0.832 | -0.015(-0.197 to 0.167) | 0.871 |
| Parent PHQ-8 | **0.182(0.085 to 0.279)** | **0.000*** | **0.148(0.053 to 0.242)** | **0.002*** |
